# Age-Related Impairment of Ultrasonic Vocalization in Tau.P301L Mice: Possible Implication for Progressive Language Disorders

**DOI:** 10.1371/journal.pone.0025770

**Published:** 2011-10-12

**Authors:** Clément Menuet, Yves Cazals, Christian Gestreau, Peter Borghgraef, Lies Gielis, Mathias Dutschmann, Fred Van Leuven, Gérard Hilaire

**Affiliations:** 1 Maturation, Plasticity, Physiology and Pathology of Respiration, Unité Mixte de Recherche 6231, Centre National de la Recherche Scientifique, Université de la Méditerranée, Université Paul Cézanne, Marseille, France; 2 Neurovegetative physiology laboratory, Unité Mixte de Recherche 6231, Centre National de la Recherche Scientifique, Université de la Méditerranée, Université Paul Cézanne, Marseille, France; 3 Experimental Genetics Group, Department of Human Genetics, Katholieke Universiteit Leuven, Leuven, Belgium; 4 Institute of Membrane and Systems Biology, University of Leeds, Leeds, United Kingdom; The University of Sydney, Australia

## Abstract

**Background:**

Tauopathies, including Alzheimer's Disease, are the most frequent neurodegenerative diseases in elderly people and cause various cognitive, behavioural and motor defects, but also progressive language disorders. For communication and social interactions, mice produce ultrasonic vocalization (USV) via expiratory airflow through the larynx. We examined USV of Tau.P301L mice, a mouse model for tauopathy expressing human mutant tau protein and developing cognitive, motor and upper airway defects.

**Methodology/Principal Findings:**

At age 4–5 months, Tau.P301L mice had normal USV, normal expiratory airflow and no brainstem tauopathy. At age 8–10 months, Tau.P301L mice presented impaired USV, reduced expiratory airflow and severe tauopathy in the periaqueductal gray, Kolliker-Fuse and retroambiguus nuclei. Tauopathy in these nuclei that control upper airway function and vocalization correlates well with the USV impairment of old Tau.P301L mice.

**Conclusions:**

In a mouse model for tauopathy, we report for the first time an age-related impairment of USV that correlates with tauopathy in midbrain and brainstem areas controlling vocalization. The vocalization disorder of old Tau.P301L mice could be, at least in part, reminiscent of language disorders of elderly suffering tauopathy.

## Introduction

Tauopathies, including Alzheimer's Disease (AD), are the most prevalent neurodegenerative disorders in elderly people and are characterized by defective learning and memory, besides other cognitive and behavioural symptoms. Tauopathies are accompanied by problems with swallowing, breathing and language. Swallowing disorders, with aspiration of foreign objects often result in pneumonia, a major cause of death in AD [1]–[3]. Sleep-disordered breathing, with obstructive apnoeas and subsequent hypoxic events possibly contributes to altered brain oxygenation and function in AD [4]–[8]. Problems with speech and language also develop during AD and in many other neurodegenerative diseases [9]–[13]. Although clinical and neuroanatomical correlates of progressive language disorders are not well understood, they are often associated with tauopathy-induced alterations of synaptic processes in forebrain networks [10], [11], [13]–[16].

Herein we examined vocalization in transgenic Tau.P301L mice, a validated mouse model of tauopathy, produced in the FVB/N genetic background and with specific expression of the human mutant Tau.P301L protein in neurons [17]. From 7–8 months onwards, Tau.P301L mice develop brain tauopathy, cognitive and motor disorders, but also upper airway dysfunction and thereafter breathing defects leading to premature death at 10–12 months [17]–[20]. For communication and social interactions, mice use ultrasonic vocalization (USV) produced by expiratory airflow through the larynx [21]–[23]. Different USV patterns, possibly representing different lexicons, behaviours or innate variations in vocal repertoires have been reported in genetically distinct mouse strains [21]–[26]. We report here for the first time a drastic age-related impairment of USV in transgenic Tau.P301L mice that correlates well with their upper airway dysfunction, reduced expiratory airflow and tauopathy in midbrain and brainstem areas controlling vocalization.

## Methods

### Ethics Statement

The experiments were performed on adult mice housed with food and water *ad libitum*, and in accordance with French national legislation (JO 87-848) and European Communities Council Directive (22 September 2010, 2010/63/EU, 74). All animal protocols were approved by our local ethics committee named “Direction Départementale de la Protection des Populations, Préfecture des Bouches du Rhône” (France), with permit numbers A13-505, 13-47 and 13-227 delivered to C. Menuet, Y. Cazals and C. Gestreau, respectively. The procedures for genetic analysis, plethysmography and histology were already reported in detail [17]–[20].

### Animals

Transgenic Tau.P301L mice were produced in the FVB/N genetic background [17], [18]. They expressed the longest human tau isoform bearing the P301L mutation (Tau.4R/2N-P301L) under control of the mouse thy1 gene promoter aiming for neuron-specific expression starting in the third postnatal week. We used obligate litters (homozygous Tau.P301L males and females; FVB/N males and females). Transgenic Tau.P301L mice were therefore homozygous for the Tau-P301L transgene and were genotyped by PCR and qPCR. They were compared with age- and sex-matched wild-type FVB/N mice as controls. Mouse rearing environments were similar for both genotypes [21]. Mice were studied at two different ages (different mice at different ages): at 4–5 months because that is pathologically pre-symptomatic and at age 8–10 months, which is in the pathological phase with progressive motor defects, clasping, brain tauopathy, loss of body-weight and breathing defects [17], [19].

### USV recordings

USV recordings were performed in a custom-build, double-walled concrete acoustic chamber. Conscious, unrestrained mice were placed in clean rectangular polyethylene cages (29×18×12.5 cm) covered by a metal wire lid. A free field microphone (type 4191, Bruel & Kjaer, Denmark) was placed 2 cm above the metal lid, in the centre of the cage. The microphone signal was sampled through audio chip (SoundMax Integrated HD) and dedicated software ( Adobe Audition 1.5) at a rate of 192 kHz allowing the recording of the frequency range 10 to 90 kHz, corresponding to the USV range of adult mice [22], [27].

**Figure 1:**
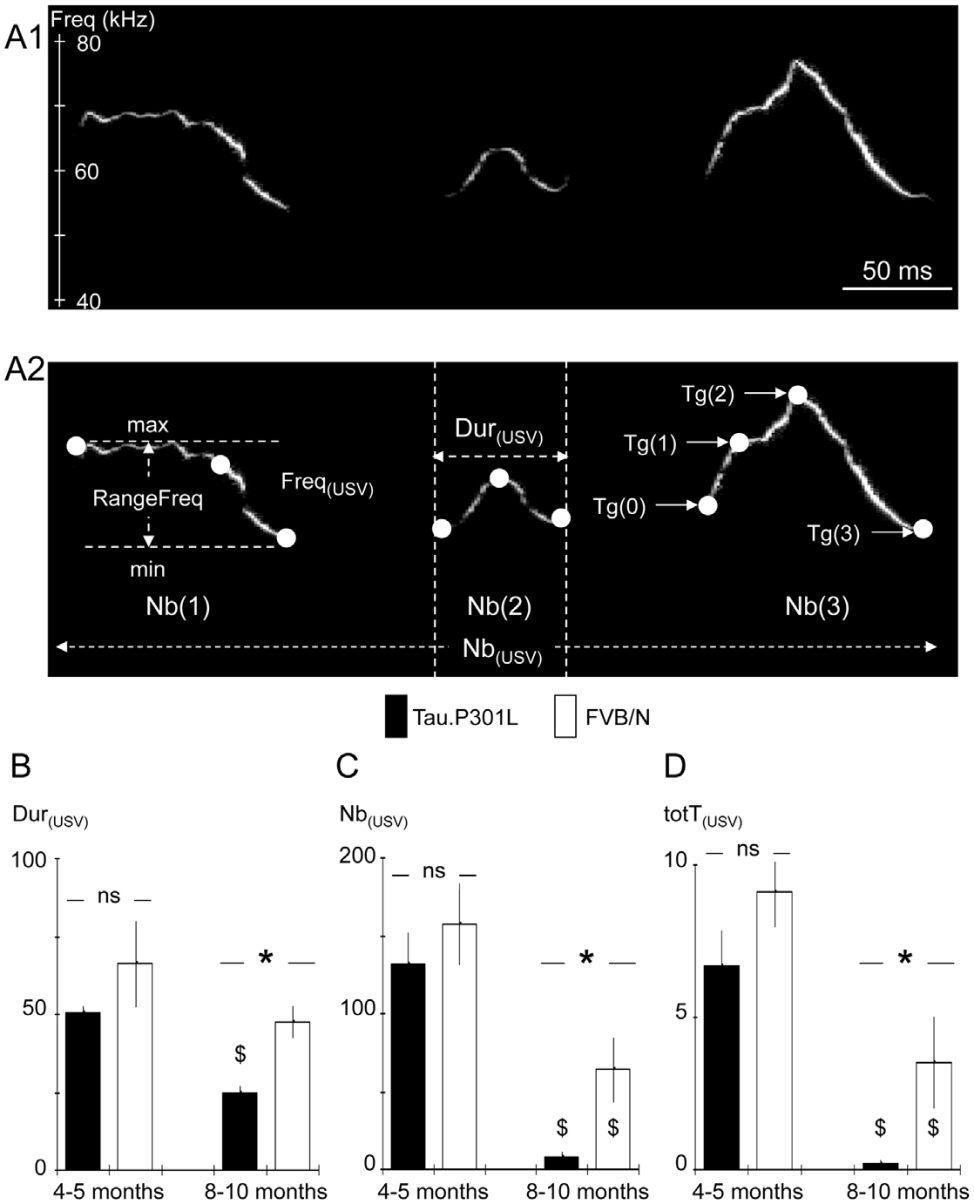
Altered USV production in old Tau.P301L mice. A1- Rough USV spectrographic display with frequency and time scales in kHz and ms, respectively. A2 – As above but showing the tags (Tg, white dots) placed at slope changes in frequency on the rough USV and the analyzed USV parameters: duration of USV (Dur_(USV)_), frequency at tags (Freq_(USV)_), max and min Freq_(USV)_ (RangeFreq), complexity (number of segments delimited by tags), and number of USV produced per min of recording (Nb_(USV)_). The total time of USV per min (totT_(USV)_) was obtained by summation of individual Dur_(USV)_. B – Columns in histograms show Dur_(USV)_ (expressed in ms) in Tau.P301L (black columns) and FVB/N (white columns) mice at age 4–5 months and 8–10 months (young and old mice, respectively). Note the significant reduction of Dur_(USV)_ in old Tau.P301L mice. C – As in B but for Nb_(USV)_ (expressed in USV per min). Note the drastic reduction of Nb_(USV)_ in old Tau.P301L mice. D – As in B but totT_(USV)_ (expressed in s per min of recording). Note the significant and drastic reduction of totT_(USV)_ in old Tau.P301L mice. * indicates a significant inter-strain difference at a given class of age and $ a significant age-related difference for a given strain; ns, non significant inter-strain difference.

**Figure 2:**
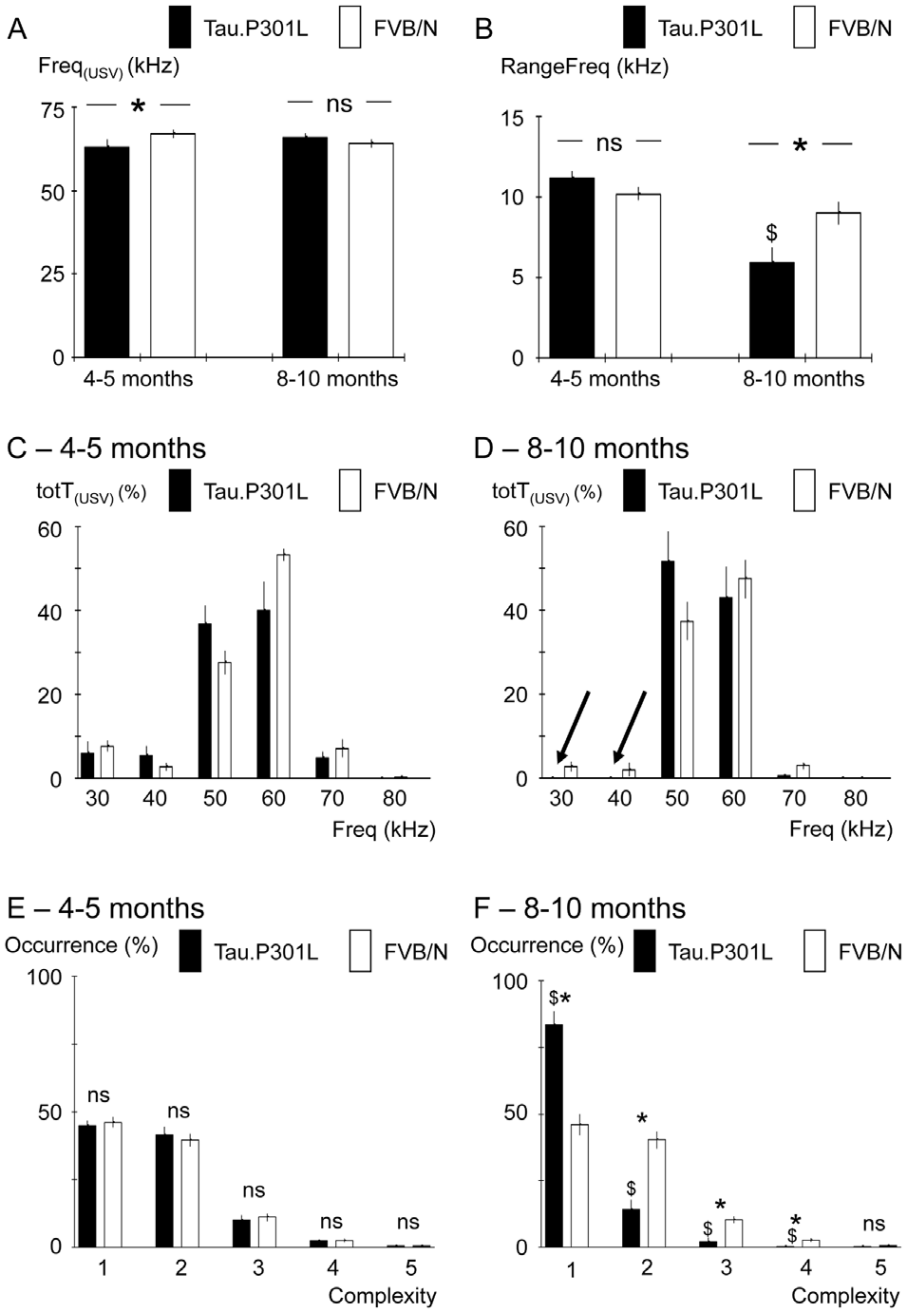
Altered USV pattern in old Tau.P301L mice. A – Columns in histograms show Freq_(USV)_ (expressed in kHz) used to produce USV in Tau.P301L (black columns) and FVB/N (white columns) mice at age 4–5 and 8–10 months. Note Freq_(USV)_ was similar in old Tau.P301L and FVB/N mice. B – As in A, but for RangeFreq ( = max Freq_(USV)_−min Freq_(USV)_, see Fig. 1A2). Note RangeFreq of old Tau.P301L mice was significantly reduced when compared to that of old FVB/N and that of young Tau.P301L mice. C - Columns in histograms show the distribution of totT_(USV)_ (in %) vs. frequency (Freq), expressed in class of 10 kHz from 30 to 80 kHz in young Tau.P301L and FVB/N at age 4–5 months. Note that most USV used high 50–60 kHz frequency but some used low 30–40 kHz frequency in both genotypes. D – As in C, but for 8–10 months old mice. Arrows highlight that old Tau.P301L mice never used the low 30–40 kHz frequency component in their USV whereas old FVB/N mice still used both low and high frequencies. E - Columns in histograms show the occurrence (%) of USV of different complexity level as defined by the number of segments (see tags in Fig. 1A2) within the USV. Complexity ranged from low (1) to high (5). Note the similar distribution of complexity in Tau.P301L and FVB/N young mice. F – As in E but for old mice. Note the increased occurrence of USV of low complexity and the reduced occurrence of USV of higher complexity (>1) in old Tau.P301L mice compared to old FVB/N and young Tau.P301L mice. Complexity of old FVB/N mice did not change when compared to that of young FVB/N mice. * indicates a significant inter-strain difference at a given class of age and $ a significant age-related difference for a given strain; ns, non significant inter-strain difference.

The experimental paradigm consisted in placing three mice of the same age and genotype into the USV recording cage. Each group of three mice consisted of one male and two females that had no interactions prior to the recording session, and each group was considered as n = 1 for statistics. Male and female mice were not sexually naïve, female oestrus phase was not checked and no attempts were made to distinguish between male and female USV. USV production was maximal at the beginning of the recording session and thereafter markedly decreased while time elapsed; we therefore stopped the recording session after 6 minutes. Data were stored and analysed off line in a blind manner. The detection of USV was performed visually using a custom program (MATLAB based) with a spectrographic display and plotting USV as frequencies (kHz) vs. time (Fig. 1A1). Using a graphic pointer on the spectrograms, each USV emission was tagged at its beginning and its end, and also at each slope change to mark its different frequency glide segments (Fig. 1A2). Based on these coordinates (Time/Frequency), different USV parameters were calculated (Fig. 1 and Fig. 2): 1) the duration of USV (Dur_(USV)_, expressed in ms), 2) the number of USV produced per min of recording (Nb_(USV)_), 3) the mean frequency at all tags within the USV (Freq_(USV)_, expressed in kHz), 4) the Freq_(USV)_ range within the USV (RangeFreq, expressed in kHz), i.e. the difference between max Freq_(USV)_ and min Freq_(USV)_ between tags, and 5) the complexity, i.e., the number of segments composing the USV (ranging from 1 to 5 for low and high complexity, respectively). The total time of USV emission (totT_(USV)_, expressed in s per min of recording) was obtained by summation of individual Dur_(USV)_ and the distribution of totT_(USV)_ vs. Freq_(USV)_ was analyzed.

To the best of our knowledge, no data are available in the literature about olfaction, hearing and vision of 8–10 months old Tau.P301L mice. Visual observations of behavioural responses of Tau.P301L and FVB/N mice to noise (click) revealed neither marked inter-strain difference nor obvious alterations of response with age, suggesting preserved hearing in old Tau.P301L mice. Individual recordings of breathing of Tau.P301L or FVB/N mice with whole-body plethysmography revealed frequent episodes of sniffing in both strains and in both age groups, suggesting preserved responses to odours in old Tau.P301L mice. Vision of FVB/N and Tau.P301L mice was not studied.

### Double-chamber plethysmography

**Figure 3:**
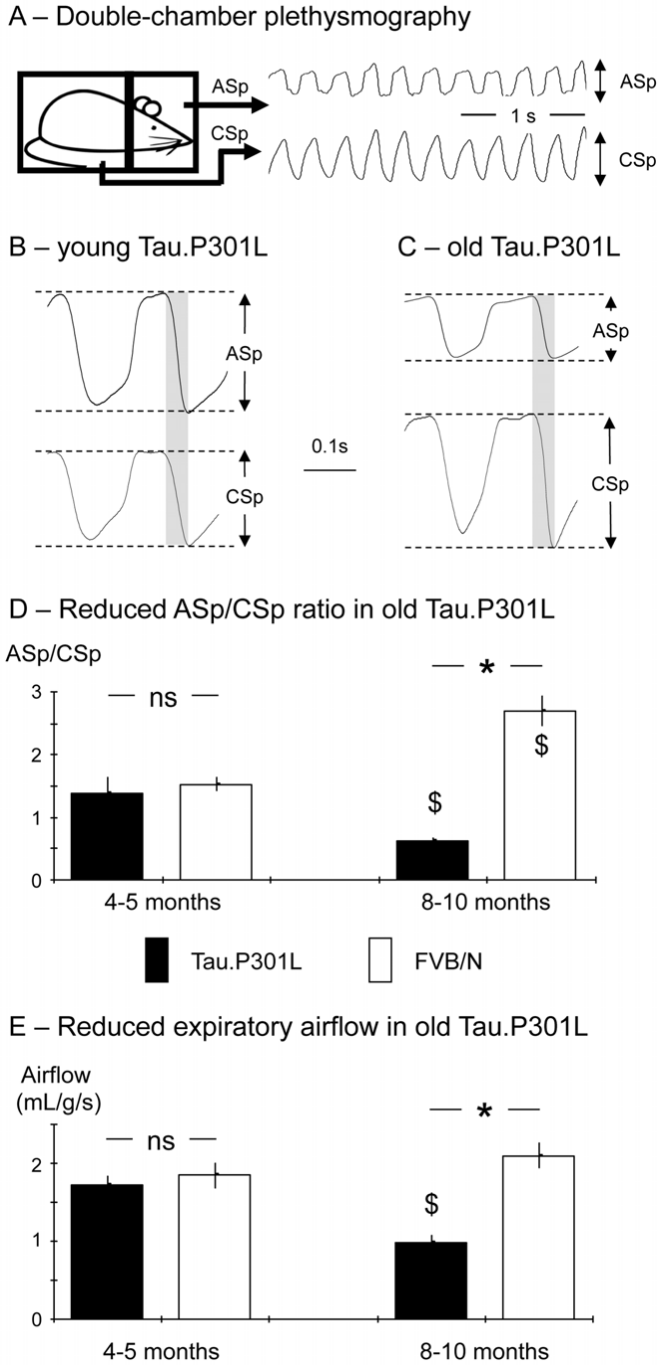
Reduced expiratory airflow in old Tau.P301L mice. A - Schematic presentation of the double-chamber plethysmographic set-up allowing the simultaneous recordings of chest spirogram (CSp; in the body chamber) and airflow spirogram (ASp; in the head chamber) in conscious mice. B, C – Averaging of about 100 successive respiratory cycles during quiet period of breathing in young (B) and old (C) mice allowed the measurements of mean ASp and CSp, the calculation of the ASp/CSp ratio and the measurement of expiratory airflow during lung emptying period (gray areas). D - Columns in histograms show the ASp/CSp ratio in Tau.P301L (black columns) and FVB/N (white columns) young and old mice. Note 1) the ratio was similar in young Tau.P301L and FVB/N mice, and 2) the ratio was significantly reduced and increased in old Tau.P301L and FVB/N mice, respectively. E – As in D but for the expiratory airflow. Note the expiratory airflow was similar in young Tau.P301L and FVB/N mice, significantly halved in old Tau.P301L mice and unchanged in old FVB/N mice. * indicates a significant inter-strain difference at a given class of age and $ a significant age-related difference for a given strain; ns, non significant inter-strain difference.

We used constant flow, double-chamber plethysmography to examine breathing parameters and upper airway function of young (4–5 months) and old (8–10 months) conscious mice [19], [20]. We simultaneously recorded the chest respiratory movements in the body chamber (Chest Spirogram, CSp) and the resulting airflow in the head chamber (Airflow Spirogram, ASp) (Fig. 3A). Small air volumes were injected within the head chamber and body chamber for checking proper sealing of chambers and for calibration purposes. To minimize stress, the mice were habituated to the plethysmograph chamber before the recording sessions. Spirograms were recorded, stored and analyzed afterwards, using only periods with stable breathing frequency. We calculated the ASp/CSp ratio; a reduced ASp vs. an increased CSp resulting in ratio <1 was considered as indicative of upper airway dysfunction impairing the chest respiratory movement to produce adequate airflow [19], [20]. After averaging of about 100 respiratory cycles, we measured the mean expiratory airflow (expressed in µl per g per second, µL/g/s). We also measured the mean respiratory frequency (expressed in cycles per min) and the duration of inspiratory and expiratory periods (expressed in ms).

### Immunohistochemistry

After anaesthesia (Nembutal; 120 mg/kg, i.p.) and transcardiac perfusion (ice-cold saline 5 ml/min, 8 min; paraformaldehyde 2 ml/min, 10 min), brain and brainstems were removed, post fixed overnight (4% paraformaldehyde), and stored (0.1% sodium azide in PBS at 4°C) until sectioning (40 µm coronal vibratome sections). Briefly, the following procedures were performed [17]–[20]: rinsing (PBS; 15 min with 1.5% H2O2 in 50% methanol/PBS), blockade of non-specific binding sites (10% fetal calf serum, 0.1% Triton X-100 in PBS), incubation with primary monoclonal antibodies AT8 (mouse anti-AT8, 1/2500) or AT100 (mouse anti-AT100, 1/1400), 1 h incubation with the secondary goat antimouse IgG antiserum coupled to PAP (1∶500 in blocking buffer; Dako), 5 min incubation in 50 mM Tris HCl, pH 7.6, enzymatic staining with 3,3′ -diaminobenzidine (0.5 mg/ml), 0.3%H2O2 in 50 mM Tris HCl, pH 7.6. Sections were counterstained with hematoxylin, ethanol dehydrated, delipidated in xylol and mounted for microscopic analysis of AT8 and AT100 immunoreactivity.

AT8 and AT100 are monoclonal antibodies specifically directed against phosphorylated human protein tau at epitopes pS198/pS202pS/pS205 and pT231/pS235, respectively (Innogenetics, Gent, Belgium). AT8 is observed in brain of young adults and children and is a marker on the border between physiology and pathology whereas AT100 is a recognized pathological phospho-epitope, typical for AD and primary tauopathies [17], [28], [29]. AT8 and AT100 expression profiles in brainstem sections were examined using neuroanatomical reference points from mouse atlas [30]. For a given mouse, about 140–150 consecutive coronal sections were cut, from about 0.5 mm caudal to the pyramidal decussation to about 5 mm more rostral. Sections were stained in groups of three with AT8 (first section), AT100 (second section) or saved (third section). In sections from old Tau.P301L mice, we counted the AT100 positively stained (AT100+) neurons, defined as neurons with visible nucleus, well-marked soma, and high staining intensity. Counting neurons twice was not possible because AT100 stained sections are at least 80 µm apart.

### Statistics

Statistical analysis of USV and breathing parameters in young and old FVB/N and Tau.P301L mice was performed using ANOVA with post-hoc Newman-Keuls test in the case of normally distributed data, or Kruskal-Wallis with post-hoc Dunn test in the case of non-normally distributed data (Igor Pro software; WaveMetrics, Oregon, USA). Values are given as mean ± standard error of the mean (S.E.M.). Statistical differences were regarded as significant if *p*<0.05.

## Results

### Ultrasonic Vocalisation (USV) disorders in old Tau.P301L mice

We analyzed USV parameters (Fig. 1A1, A2) of conscious, unrestrained and age-matched Tau.P301L and FVB/N mice. USV parameters were in the same range in young Tau.P301L and FVB/N mice (4–5 months) but differed substantially between old Tau.P301L and FVB/N mice (8–10 months) (Table 1).

**Table 1:** Main USV parameters of young and old Tau.P301L and FVB/N mice.

Mouse strain	Age	n	Dur_(USV)_	Nb_(USV)_	totT_(USV)_	Freq_(USV)_	RangeFreq
Tau.P301L	Young	18	51±2	132±24	6.7±1.1	63±2	11.2±0.4
*P(inter-strain diff.)*			*0.186*	*0.375*	*0.174*	*0.044*	*0.277*
FVB/N	Young	21	66±14	156±24	9.1±1.2	67±1	10.2±0.4
Tau.P301L	Old	18	25±2	6±0	0.2±0.1	66±1	5.9±0.9
*P(inter-strain diff.)*			*0.047*	*0.041*	*0.047*	*0.499*	*0.002*
FVB/N	Old	24	48±5	66±2	3.5±1.5	64±1	9.0±0.7
*P(inter-age diff.)Tau.P301L*			*0.037*	*<0.001*	*0.002*	*0.181*	*<0.001*
*P(inter-age diff.)FVB/N*			*0.088*	*0.002*	*0.002*	*0.157*	*0.182*

Mean ± SEM values expressed in ms for Dur_(USV)_, number USV per min for Nb_(USV)_, s per min of recording for totT_(USV)_, kHz for Freq_(USV)_ and RangeFreq; n, number of studied mice; *p* values for inter-strain (Tau.P301L vs. FVB/N) and inter-age (young vs. old) comparisons are considered significant when p<0.05.

In young mice, no significant differences were observed in either the duration of individual USV (Dur_(USV)_; Fig. 1B), or the number of USV produced per min (Nb_(USV)_; Fig. 1C), or the total amount of time spent in producing USV (totT_(USV)_; Fig. 1D). To produce USV, both Tau.P301L and FVB/N young mice used a mean frequency of about 65 kHz (Freq_(USV)_; Fig. 2A). However, Freq_(USV)_ was slightly (6%) but significantly lower in Tau.P301L than FVB/N mice (Table 1). Within the USV, the range of Freq_(USV)_ (RangeFreq = max Freq_(USV)_−min Freq_(USV)_ ) was similar in Tau.P301L and FVB/N mice (Fig. 2B) as well as the distribution of totT_(USV)_ within the different classes of Freq_(USV)_ (Fig. 2C). Young Tau.P301L and FVB/N mice preferentially used high 50–60 kHz Freq_(USV)_, and occasionally the low 30–40 kHz Freq_(USV)_. We also analyzed the USV complexity by counting the number of segments within USV (tags in Fig. 1A2). In both strains (Fig. 2E), most USV had low complexity (1 or 2 segments) with only some of higher complexity (>2 segments).

In the old mice, significant differences were observed, with shorter, more rare and much simpler USV in transgenic Tau.P301L mice than in wild-type FVB/N mice (Table 1). In Tau.P301L mice, the duration of USV decreased with age and Dur_(USV)_ became significantly 2-fold shorter in old relative to young Tau.P301L mice (Fig. 1B). In FVB/N mice, Dur_(USV)_ did not significantly decrease with age, in contrast to the significant 2-fold reduction in old Tau.P301L mice. The number of USV Nb_(USV)_ decreased with age in both strains (Fig. 1C), but the Nb_(USV)_ reduction was dramatic in Tau.P301L mice (20-fold reduction) versus modest in wild-type FVB/N mice (2-fold reduction). Not surprisingly, the substantial reductions of both Dur_(USV)_ and Nb_(USV)_ in old Tau.P301L mice dramatically reduced totT_(USV)_ (Fig. 1D): i.e. 30-fold weaker in old than in young Tau.P301L mice. On the other hand, totT_(USV)_ was also significantly reduced in old FVB/N mice but the reduction was modest in FVB/N mice when compared to that of Tau.P301L mice: totT_(USV)_ was 15-fold weaker in old Tau.P301L than in old FVB/N mice. In addition, the USV pattern became simpler and more monotonous in old Tau.P301L than in old FVB/N mice, while the preferential 65 kHz Freq_(USV)_ remained nearly unchanged (Fig. 2A). In old Tau.P301L mice, the RangeFreq was significantly reduced compared to young Tau.P301L mice and to old FVB/N mice (Fig. 2B). Plotting totT_(USV)_ vs. Freq_(USV)_ revealed that old Tau.P301L mice never used the low 30–40 kHz Freq_(USV)_ they occasionally used at younger age, whereas old FVB/N mice still used both high and low Freq_(USV)_ (arrows in Fig. 2D). In addition, the USV complexity became significantly reduced in old Tau.P301L mice (Fig. 2F), with a doubling of the number of the simplest USV (only one segment) versus only half the number of the more complex USV (2 and 3 segments). In contrast, the USV complexity did not change in aged FVB/N mice.

In summary, old Tau.P301L mice developed a drastic impairment of USV meanwhile wild-type FVB/N mice only showed a modest reduction in the number of USV with aging.

### Upper Airway dysfunction reduces expiratory airflow in old Tau.P301L mice

USV are produced by expiratory airflow through the larynx [31]–[34]. From 7–8 months onwards, Tau.P301L mice develop upper airway dysfunction and abnormal expiratory laryngeal activity [19], which may significantly affect their USV.

We therefore measured the expiratory airflow of conscious, young and old transgenic Tau.P301L mice and wild-type FVB/N mice (Table 2). We used double-chamber plethysmography (Fig. 3A) to record the chest spirogram (CSp) produced by the chest respiratory movements in the body chamber and the resulting airflow spirogram (ASp) in the head chamber. We calculated the ASp/CSp ratio, an index of upper airway function [19]. Young Tau.P301L and FVB/N mice had similar ASp/CSp ratio >1, indicative of correct upper airway function (Fig. 3B, 3D). Measuring the expiratory airflow revealed similar values in young Tau.P301L and FVB/N mice (Fig. 3E). In old mice however, significant differences were observed with reduced ASp and increased CSp in old Tau.P301L mice (Fig. 3C) but not in old FVB/N mice. Moreover, the ASp/CSp ratio was significantly reduced in old Tau.P301L mice (Fig. 3D) but not in old FVB/N mice where it was even significantly increased (Table 2). The ASp/CSp ratio <1 in old Tau.P301L mice corroborated their upper airway dysfunction [19], [20]. The expiratory airflow was significantly reduced in old Tau.P301L mice but not in old FVB/N mice (Fig. 3E): it became 2-fold weaker in old Tau.P301L mice relative to young Tau.P301L mice, and to young and old FVB/N mice (Table 2). Despite their upper airway dysfunction, old Tau.P301L mice retained normal respiratory frequency, duration of inspiratory period and duration of expiratory period when compared to the other three groups of mice (Table 2).

**Table 2:** Main breathing parameters of young and old Tau.P301L and FVB/N mice.

Mouse strain	Age	n	ASp/CSp	Exp Airflow	Rf	Ti	Te
Tau.P301L	Young	8	1.39±0.24	1.72±0.11	207±9	155±14	149±11
*P(inter-strain diff.)*			*0.356*	*0.274*	*0.259*	*0.266*	*0.168*
FVB/N	Young	6	1.53±0.11	1.85±0.16	201±12	169±26	172±36
Tau.P301L	Old	15	0.63±0.4	0.97±0.09	193±10	176±13	142±7
*P(inter-strain diff.)*			*<0.001*	*<0.001*	*0.207*	*0.405*	*0.233*
FVB/N	Old	20	2.71±0.22	2.10±0.16	208±13	177±16	134±16
*P(inter-age diff.)Tau.P301L*			*<0.001*	*<0.001*	*0.221*	*0.194*	*0.266*
*P(inter-age diff.)FVB/N*			*0.004*	*0.202*	*0.482*	*0.477*	*0.085*

Mean ± SEM values expressed in mL/g/s for expiratory airflow (Exp Airflow), cycle per min for respiratory frequency (Rf), and ms for duration of inspiratory (Ti) and expiratory (Te) periods; n, number of studied mice; *p* values for inter-strain (Tau.P301L vs. FVB/N) and inter-age (young vs. old) comparisons are considered significant when p<0.05.

### Tauopathy develops in brainstem areas of old Tau.P301L mice

From 7–8 months onwards, Tau.P301L mice progressively develop brainstem tauopathy [17], [19], [20], which may affect central networks controlling upper airways and vocalization.

**Figure 4:**
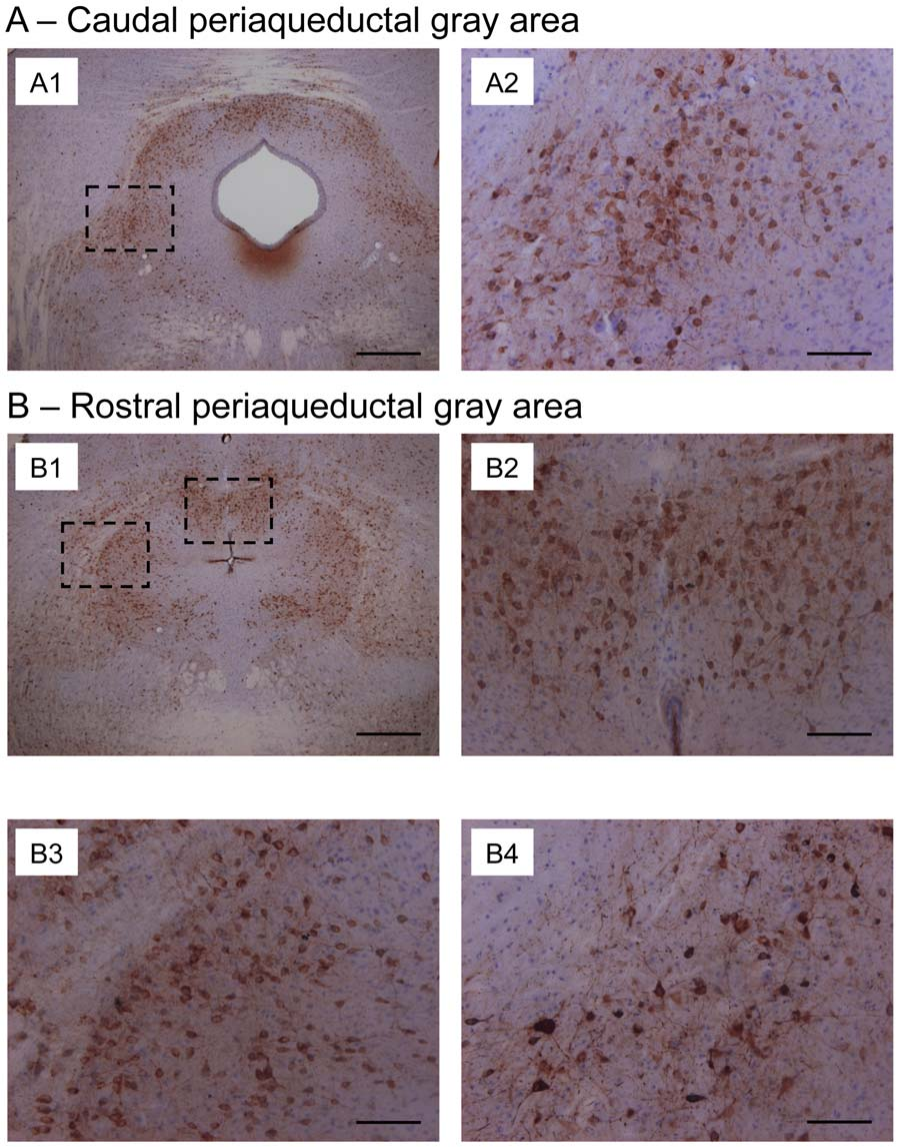
Tauopathy in the PAG of old Tau.P301L mice. Immunohistochemistry with AT100 as tauopathy marker on midbrain coronal sections of old Tau.P301L mice reveals dramatic tauopathy in the whole PAG, affecting both its caudal (A) and rostral (B) parts. A2, B2 and B3 are enlargements of the dotted line boxes drawn in A1 and B1, and show high density of AT100+ neurons in the caudal, ventro-lateral PAG (A2), the rostral, dorso-median PAG (B2) and the rostral dorso-lateral PAG (B3) of the same old Tau.P301L mouse. B4 shows frequent AT100+ neurons in the rostral, dorso-lateral PAG of another old Tau.P301L mice. Calibration bars: 500 µm for A1, B1; 100 µm for A2, B2–B4.

**Figure 5:**
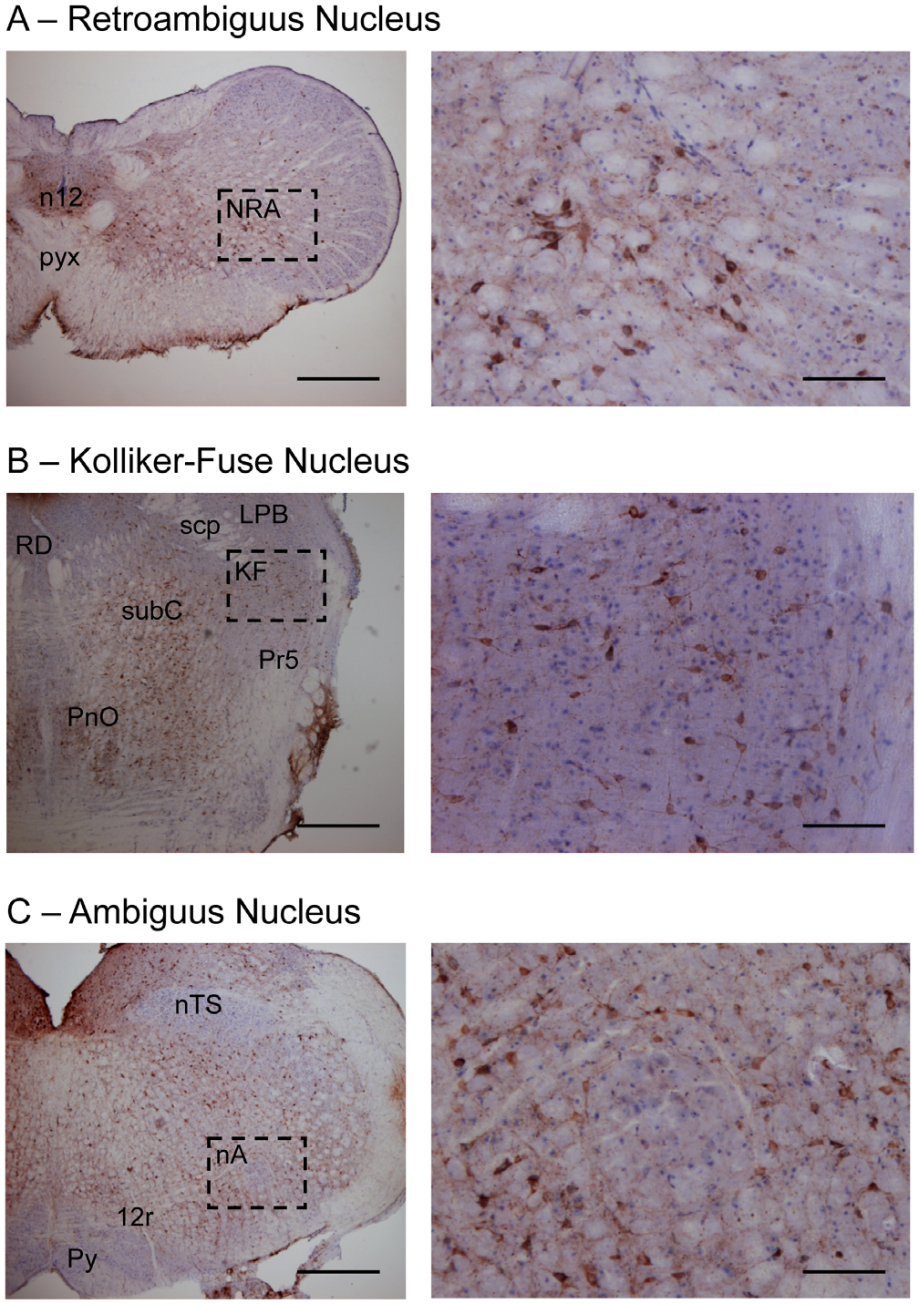
Tauopathy in the NRA and KF areas of old Tau.P301L mice. Immunohistochemistry with AT100 as tauopathy marker on brainstem coronal sections of a given Tau.P301L mouse (as in Fig. 4). Right hand pictures are enlargements of the dotted line boxes drawn in left hand pictures. Sections show that AT100+ neurons are frequent in the NRA (A) and the KF (B) but lacking in the nucleus tractus solitarius and nucleus ambiguus (C). Calibration bars: 500 and 100 µm for left and right hand pictures, respectively. Labels in sections indicate the Kolliker-Fuse nucleus (KF), lateral parabrachial nucleus (LPB), nucleus ambiguus (nA), nucleus tractus solitarius (nTS), hypoglossal motor nucleus (n12), oral pontine reticular nucleus (PnO), principal trigeminal sensory nucleus (Pr5), pyramidal tract (Py), pyramidal decussation (Pyx), superior cerebella peduncle (scp), raphé dorsalis (RD), subcoeruelus nucleus (subC) and intra-medullary rootlet of hypoglossal nerve (12r).

We therefore examined by immunohistochemistry sections of midbrain and brainstem of young and old mice using two distinct antibodies against phosphorylated tau epitopes, AT8 and AT100. AT100, a recognized marker of tauopathy, was not expressed in any of the young or old FVB/N mice (n = 3), and very weakly or practically absent in young Tau.P301L mice (n = 3), while markedly expressed in old Tau.P301L mice (n = 3), as reported previously [17]. The AT100 signal was particularly expressed in midbrain and brainstem sections of old Tau.P301L mice with most dramatic tauopathy in the midbrain periaqueductal gray (PAG), containing the highest density of AT100 positive (AT100+) neurons in the brainstem (Fig. 4). We counted around 100 well-stained AT100+ neurons per PAG section in the three studied old Tau.P301L mice. AT100+ neurons were observed at all rostro-caudal levels of the PAG and in all the PAG sub-regions, i.e. the ventro-lateral part (Fig. 4A2), the dorso-median part (Fig. 4B2) and the dorso-lateral part (Fig. 4B3). Secondly, we observed a high density of AT100+ neurons in the nucleus retroambiguus (NRA) in the caudal medulla (Fig. 5A) and in the Kolliker-Fuse (KF) nucleus in the dorso-lateral pons (Fig. 5B). AT100+ neurons delimitated well the KF area, extending between the middle and superior cerebellar peduncles, below the lateral parabrachial nucleus and above the principal sensory trigeminal nucleus (Fig. 5B). We counted around 25–50 well-stained AT100+ neurons per sections all along the whole rostro-caudal extension of the KF (about 700–1000 µm). In the caudal medulla, frequent AT100+ neurons were also found in the NRA area, previously defined in mouse brainstem [35]. We consistently counted around 10 well-stained, packed AT100+ neurons per studied section from about 500 µm caudal to 500 µm rostral to the pyramidal decussation. Asides the PAG, KF and NRA areas, AT100+ neurons were observed scattered in the whole brainstem, but were especially dense in the raphé obscurus, raphé magnus, locus coeruleus (data not shown), oral pontine reticular nucleus and subcoeruleus (Fig. 5B).

Conversely, some other areas appeared markedly spared by tauopathy. We found almost no AT100+ neurons in the nucleus ambiguus (nA) and the nucleus tractus solitarius (nTS) (Fig. 5C), the raphé dorsalis (Fig. 5B) and most of the cranial motor nuclei (data not shown). The hypoglossal motor nucleus was also generally spared by tauopathy but some AT100+ neurons were observed in the caudal part of the hypoglossal nucleus of one of the three studied Tau.P301L mice (Fig. 5A). In addition, as previously reported in Tau.P301L mice at the terminal stages [20], the respiratory-related areas implicated in respiratory rhythmogenesis (in the ventral medulla below the nA) and in central chemosensitivity (in the very ventral medulla below the facial motor nucleus) did not contain significant amount of AT100+ neurons.

The epitope defined by AT8 is a marker on the border between physiology and pathology, and we confirmed its previously reported expression profile in the brainstem of old Tau.P301L mice [19], [20]. Dense AT8 expression was evident in the PAG, KF and NRA areas, and more scattered in the whole reticular formation, with increased density in the raphé obscurus and magnus, locus coeruleus (data not shown). Only rare AT8+ neurons were in the nA, the nTS, the hypoglossal motor nucleus (some in one mouse), the other motor cranial nuclei and the respiratory-related areas of the ventral medulla (data not shown).

To summarize, both AT100 and AT8 revealed severe tauopathy in old Tau.P301L mice, especially affecting the midbrain PAG, pontine KF and medullary NRA areas, known to control upper airway and vocalization.

## Discussion

Compelling evidence exists that mice produce USV for communications and social interactions [21]–[23] and that mouse models of Angelman Syndrome [24], autism [25] or AD [26] produce specific USV. However, these aspects have been studied mainly in young dams and pups, not in aging mice. In the aging Tau.P301L mouse model of tauopathy [17], [18], we report for the first time a dramatic age-related impairment of USV which may be, at least in part, reminiscent of progressive language disorders of elderly people suffering tauopathy and neurodegenerative diseases.

### USV impairment of Tau.P301L mice is linked to upper airway dysfunction

Respiration and vocalization are two tightly linked motor acts that implicate the same groups of chest, abdominal and upper airway muscles. USV emission originates from expiratory airflow through the larynx as demonstrated by larynx excision, tracheotomy and motor nerve transection [31]–[34]. The motor neurons controlling the laryngeal muscles belong to the nA, with intermingled dilatator and constrictor motor neurons. They are multi-functional neurons driven by several central pattern generators, including those for respiration, vocalization and swallowing [36]. During USV, the inspiratory dilatator nA neurons become silent meanwhile the expiratory constrictor nA neurons are activated, mostly prior to USV [37], [38].

From 7–8 months onwards, Tau.P301L mice develop upper airway dysfunction [19]: an inspiratory shift of the period of activity of expiratory laryngeal motor neurons induces a paradoxical tendency to laryngeal closure during inspiration, which subsequently reduces air entry within the lungs. Consistent with these previous results, we report here that the expiratory airflow is halved in old Tau.P301L mice. Thus the abnormal laryngeal motor activity and the expiratory airflow reduction highly likely contribute to reduce the ability of old Tau.P301L mice to produce frequent, long-lasting and complex USV. Conversely, old wild-type FVB/N mice retain normal expiratory laryngeal discharge [19], normal expiratory airflow and rather spared USV, with only a modest reduction of Nb_(USV)_ and totT_(USV)_. As discussed below with AT100 expression, the impairments of laryngeal discharge, expiratory airflow and USV production in old Tau.P301L mice do not originate from a direct alteration of laryngeal motor neurons but result from alteration of their central drivers.

### USV impairment of Tau.P301L mice originates from PAG, KF and NRA tauopathy

**Figure 6:**
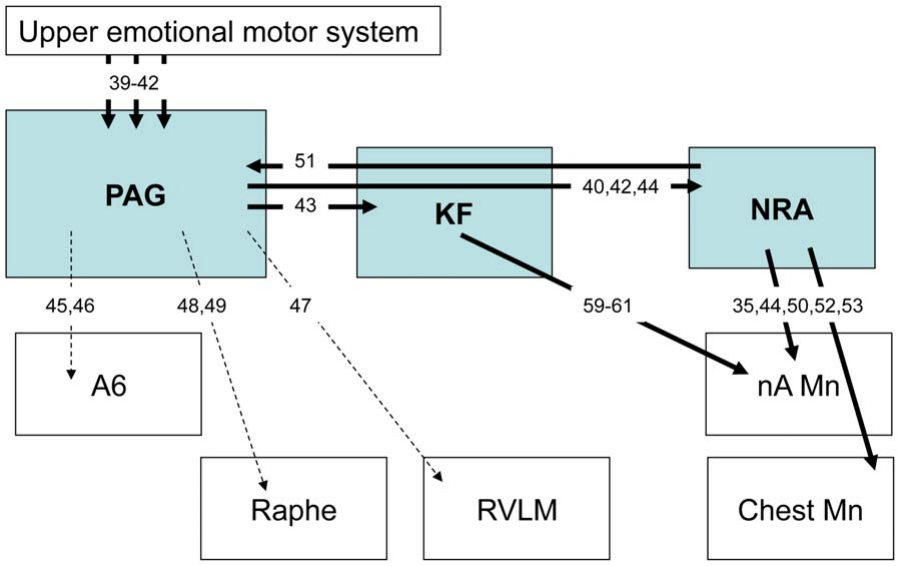
Summary diagram of the organization of the PAG, KF and NRA network controlling vocalization. Arrows indicate the demonstrated connections between structures controlling vocalization and numbers within arrows the related publications in the reference list. Mn, motor neurons; A6, locus coeruleus; RVLM, rostral ventrolateral medulla.

We previously reported that Tau.P301L mice develop brainstem tauopathy from 7–8 months onwards, with frequent AT8+ neurons in the KF revealing an altered control of upper airway function [17], [19]. Here, we used the late tau pathological marker AT100 to confirm the KF alteration in old Tau.P301L mice and reported numerous AT100+ neurons in the pontine KF but also the midbrain PAG and medullary NRA nuclei. These three structures are crucial for the control of upper airway function and vocalization as illustrated in the summary diagram of Fig. 6. The PAG is the main descending relay of the emotional motor system which converts higher emotional and cognitive commands into motor activity for complex behaviours, including respiration, vocalization and copulation [39]–[42]. The PAG projects to both the KF [43] and the NRA [40], [42], [44]. It also targets many other structures such as the locus coeruleus [45], [46], the rostro ventromedial medulla [47] and the raphé magnus [48], [49]. The PAG does not directly control the chest, abdominal and upper airway motor neurons but uses the NRA as a relay [50] and the NRA in turn projects to the PAG [51]. The NRA contains multifunctional pre-motoneurons that target the laryngeal nA motor neurons and the thoraco-abdominal motor neurons [35], [44], [50], [52], [53] and controls their activity during respiration [54], vocalization [42], [50], [55], coughing, sneezing [56], [57] and copulation [42], [58]. The KF modulates the activity of laryngeal and tongue motor neurons [59]–[61], controlling the upper airway function [19], the expression of learned upper airway behaviours [62] and the vocal patterning [63].

We propose that tauopathy-induced alterations of the crucial PAG, KF and NRA networks play a major role in the USV impairment of old Tau.P301L mice, while not excluding the implication of additional networks. Currently, no data are available in the literature on olfaction, hearing and vision of 8–10 months old Tau.P301L mice and we have observed no obvious defects in sniffing and hearing that could explain their USV impairments. Old Tau.P301L mice develop tauopathy in cortical and thalamic areas [17], which may impact on their social interactions, emotional status and subsequently USV, possibly via the PAG, KF and NRA relays. Old Tau.P301L mice also develop tauopathy in the locus coeruleus and some raphe nuclei, which may affect the monoaminergic modulations. USV are affected by the serotoninergic system [64] and the serotonin metabolism of Tau.P301L mice becomes abnormal at terminal stages of the disease [20]. On the other hand, AT100+ neurons are rare, almost absent in the nA, which excludes a direct alteration of nA motor neurons and reinforces the concept of indirect effects via alterations of PAG, KF and NRA networks. Similarly, AT100+ neurons are rare in the nTS where the peripheral respiratory inputs are integrated and in the ventral medulla where the respiratory rhythm is, at least in part, generated. Consistently, old Tau.P301L retain normal respiratory frequency, duration of inspiration and duration of expiration (present results) and develop marked breathing defects only at terminal stages of the disease [20].

### Translational aspects of mouse USV impairment to progressive language disorders

In mice, the different strain-specific USV patterns are viewed as different lexicons or innate variations in vocal repertoires [21]–[26]. USV are proposed as models for speech and socio-cognitive disorders [65] and for drugs and genes effects on social motivation, affect regulation and communication [21]. It is outside the scope of this study to define or speculate on the USV impairment in old Tau.P301L mice in terms of social interactions, lexicon or semantic defects. However, the possible link between the USV impairment in Tau.P301L mouse and the progressive language disorders in patients is worth noticing.

Aging from 4–5 to 8–10 months had only minor effects on USV of wild-type FVB/N mice: it significantly but modestly reduced Nb_(USV)_ and totT_(USV)_ and had no significant effect on Dur_(USV)_, RangeFreq, use of low Freq_(USV)_ components and complexity. In contrast, aging had major effects on USV of Tau.P301L mice where tauopathy not only exacerbated the modest age-related reduction of USV observed in FVB/N mice, dramatically reducing the quantitative USV parameters (Dur_(USV)_, Nb_(USV)_ and totT_(USV)_ ), but also significantly altered the qualitative USV parameters (RangeFreq, use of low Freq_(USV)_ components and complexity). Indeed, analyzing Tau.P301L at intermediate ages between 5 and 8 months might be highly informative about the link between histopathology and functional USV deficits.

In healthy humans, normal aging affects respiration and vocalization, reducing the ability to generate the required air pressure for speech production [66], [67]. Old persons initiate speech at a higher lung volume and produce fewer syllables per breath than young adults [68], [69]. These reductions of speech performance during normal aging are negligible compared to pathological language disorders occurring with tauopathy. Progressive language disorders concern a group of clinically, genetically and pathologically heterogeneous neurodegenerative disorders, with different variants based on motor speech, linguistic and cognitive features [9], [15], [70], [71]. However, neither language disorder phenotyping nor brain imaging alone appears a reliable predictor of pathology [72]. A few case reports suggest possible links between language disorder, swallowing impairment, respiratory difficulties and brainstem alterations [73]–[77]. In addition, PAG abnormalities have been reported in some cases of mutism [78], in AD [79] and possibly in frontotemporal dementia [80] and Parkinson disease [81]. But these reports are rare and atypical when compared to the plethora of reports about forebrain imaging and language disorders.

In a mouse model for tauopathy, we report an age-related impairment of vocalization accompanied with tauopathy of the PAG, KF and NRA network controlling vocalization. As it cannot be excluded that the PAG, KF and NRA network is also altered in elderly suffering tauopathy [73]–[81], we suggest that imaging studies in old patients with progressive language disorders concern not only the forebrain but also the midbrain and brainstem structures.
